# Supplementary material for: Commercial Gilthead Seabream (Sparus aurata L.) from the Mar Menor Coastal Lagoon as Hotspots of Microplastic Accumulation in the Digestive System
Source: Int J Environ Res Public Health. 2021 Jun 25;18(13):6844. doi: 10.3390/ijerph18136844 (PMC8297190; doi:10.3390/ijerph18136844)
Supplement: Supplementary file 1 [file ijerph-18-06844-s001.zip › ijerph-1259620-supplementary.pdf]

International Journal of
Environmental Research and Public Health

MDPI

Supplementary Material: Commercial Gilthead Seabream (Sparus aurata L.) from the Mar Menor Coastal Lagoon as Hotspots of Microplastic Accumulation in the Digestive System

Javier Bayo [1,*], Dolores Rojo [1], Pedro Martínez-Baños [2], Joaquín López-Castellanos [1] and Sonia Olmos [1]

1 Department of Chemical and Environmental Engineering, Technical University of Cartagena, Paseo Alfonso XIII 44, E-30203 Cartagena, Spain; drcampillo@gmail.com (D.R.); qlopezca@gmail.com (J.L.-C.); soniaespinar19@gmail.com (S.O.)
2 C&C MedioAmbiente, E-30204 Cartagena, Spain; cycmedioambiente@cycmedioambiente.com
* Correspondence: javier.bayo@upct.es

Table S1. Fish length (FL). fish weight (FW). stomach weight (SW). and intestine weight (IW) for all analyzed specimens of *Spaurus aurata*.

Sample	FL (cm)	FW (g)	SW (g)	IW (g)
1	38.00	700.00	3.57	13.76
2	38.00	750.00	4.08	18.88
3	31.00	477.00	2.83	8.73
4	32.00	450.00	1.97	6.47
5	28.50	375.00	2.87	4.73
6	28.50	343.00	1.96	3.73
7	25.50	228.90	0.98	4.10
8	24.50	208.50	0.92	2.99
9	24.50	210.00	0.92	1.68
10	27.00	289.20	1.65	6.28
11	25.00	214.10	1.21	2.63
12	31.00	413.90	1.75	9.73
13	32.00	434.40	1.90	11.62
14	40.00	737.00	2.60	18.13
15	40.50	773.00	2.49	14.66
16	40.00	789.00	2.17	19.43
17	42.00	801.00	2.64	16.15

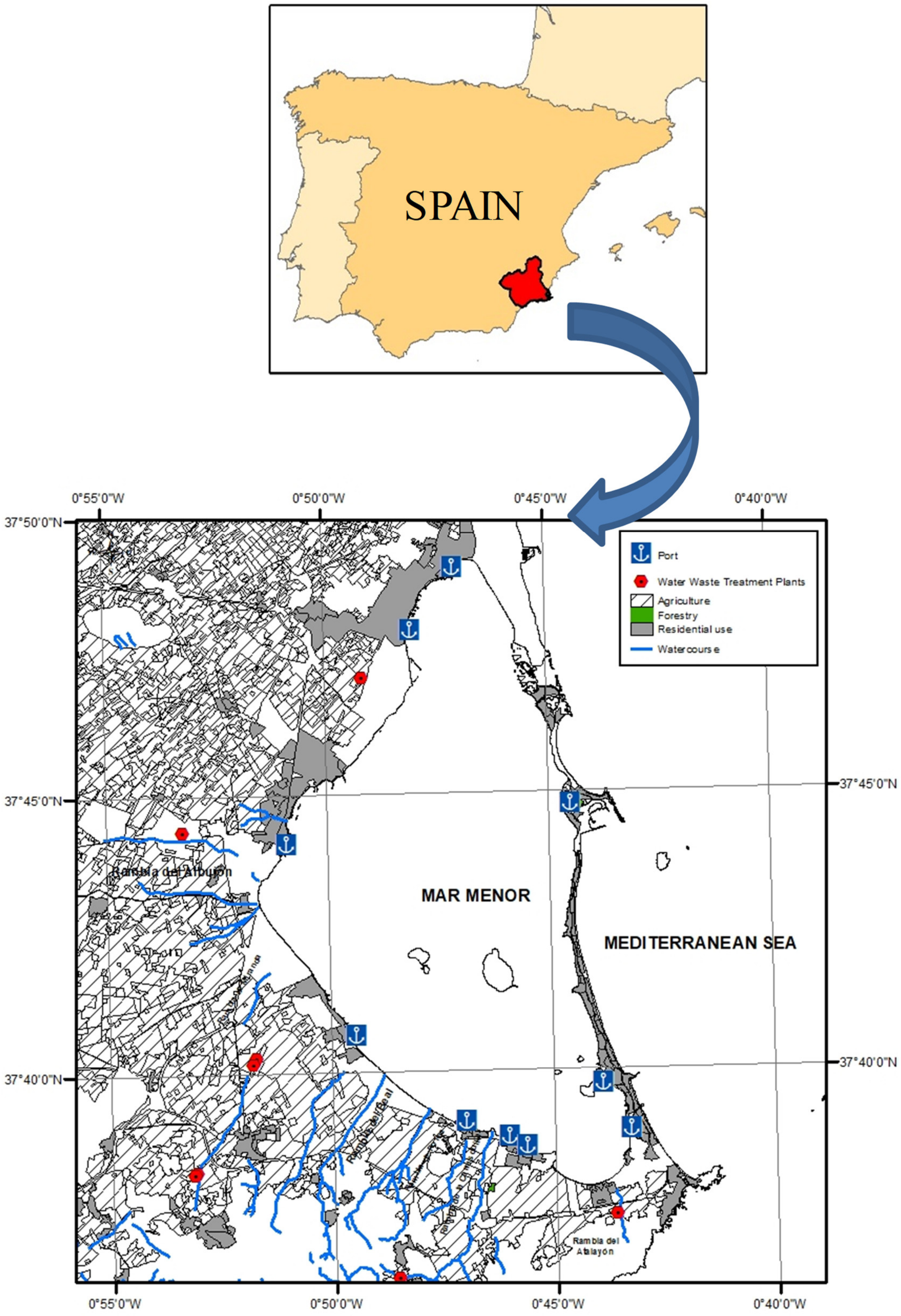

Figure S1. The Mar Menor coastal lagoon with the main fishery ports.

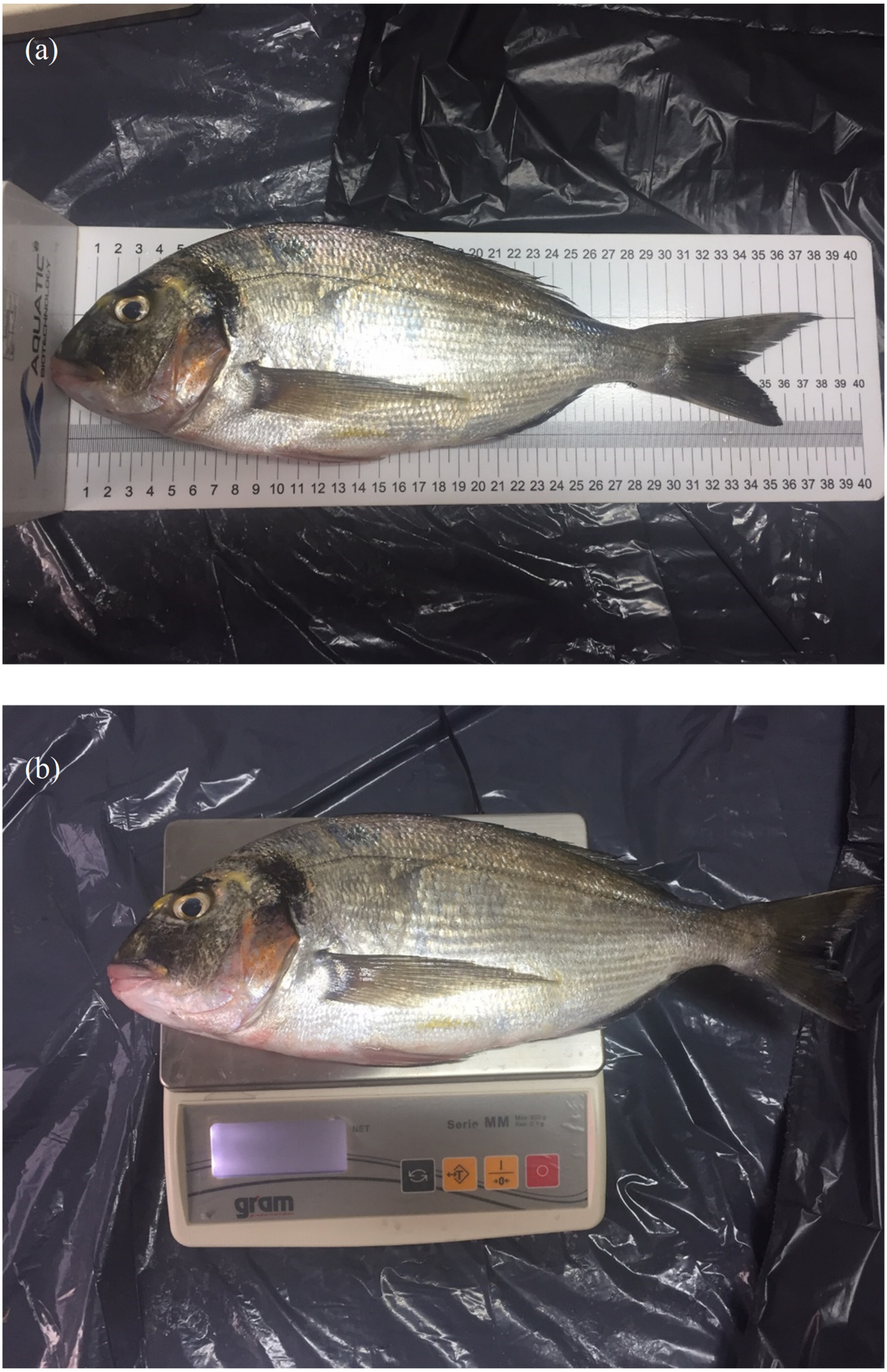

Figure S2. (**a**) Fish length, and (**b**) Fish wet weight measurements.

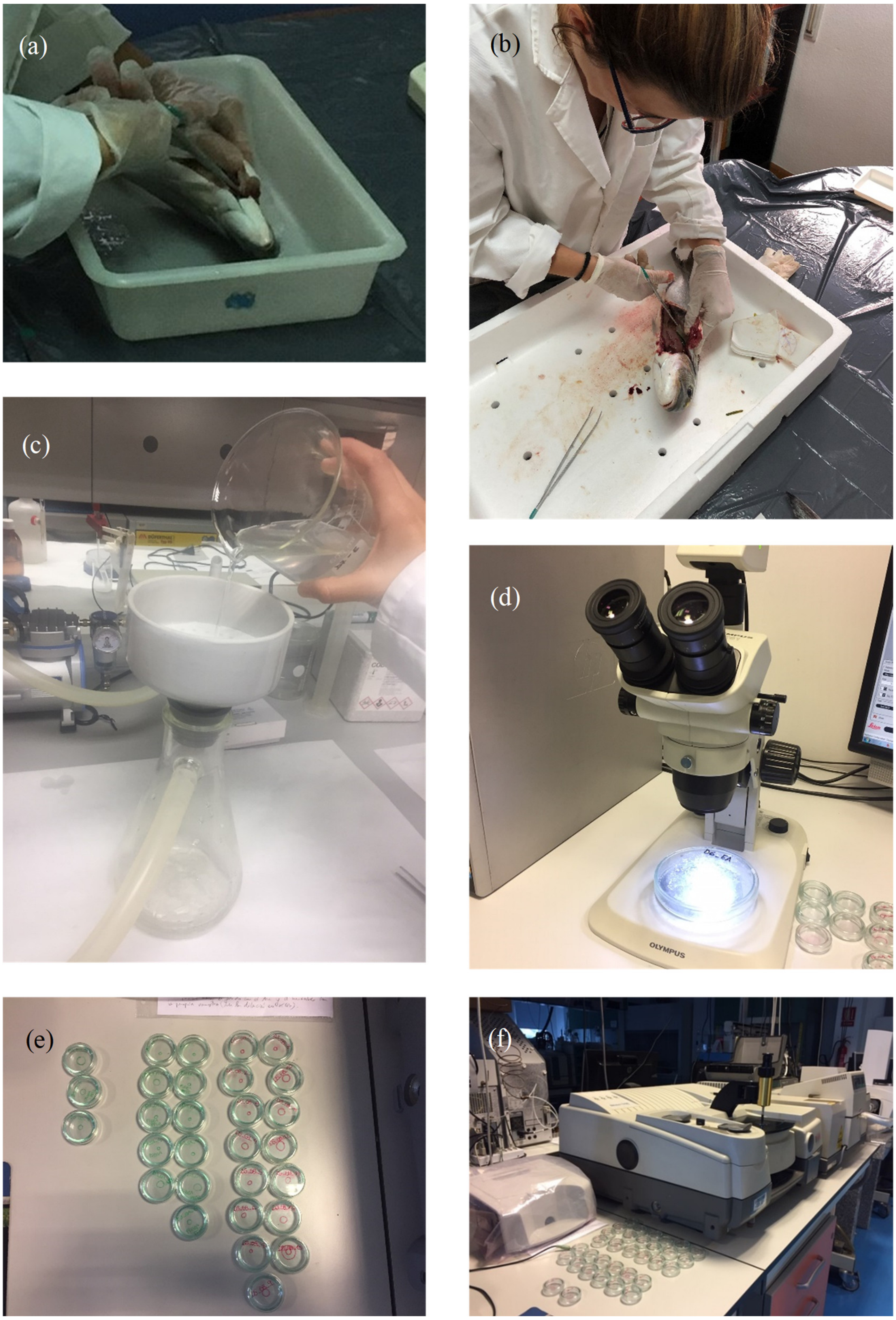

Figure S3. Sample collection and processing: (**a**) (**b**) stomach and intestine dissection; (**c**) vacuum filtration; (**d**) microscopic identification; (**e**) isolation of possible microplastics in 40-mm glass Petri Dishes; (**f**) FTIR identification.

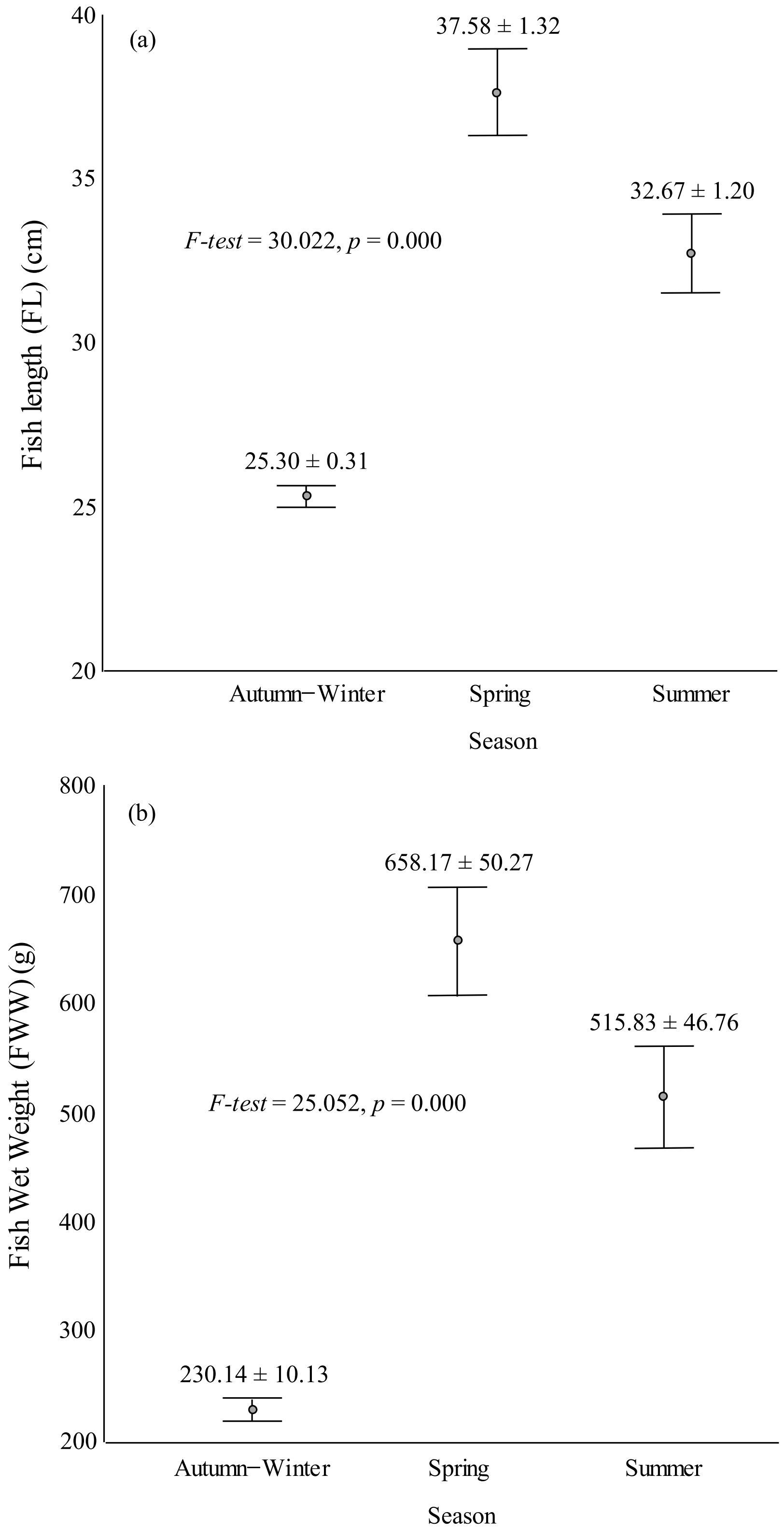

Figure S4. Average values of (**a**) Fish length (FL), and (**b**) Fish Weight (FW), according season and ANOVA tests.

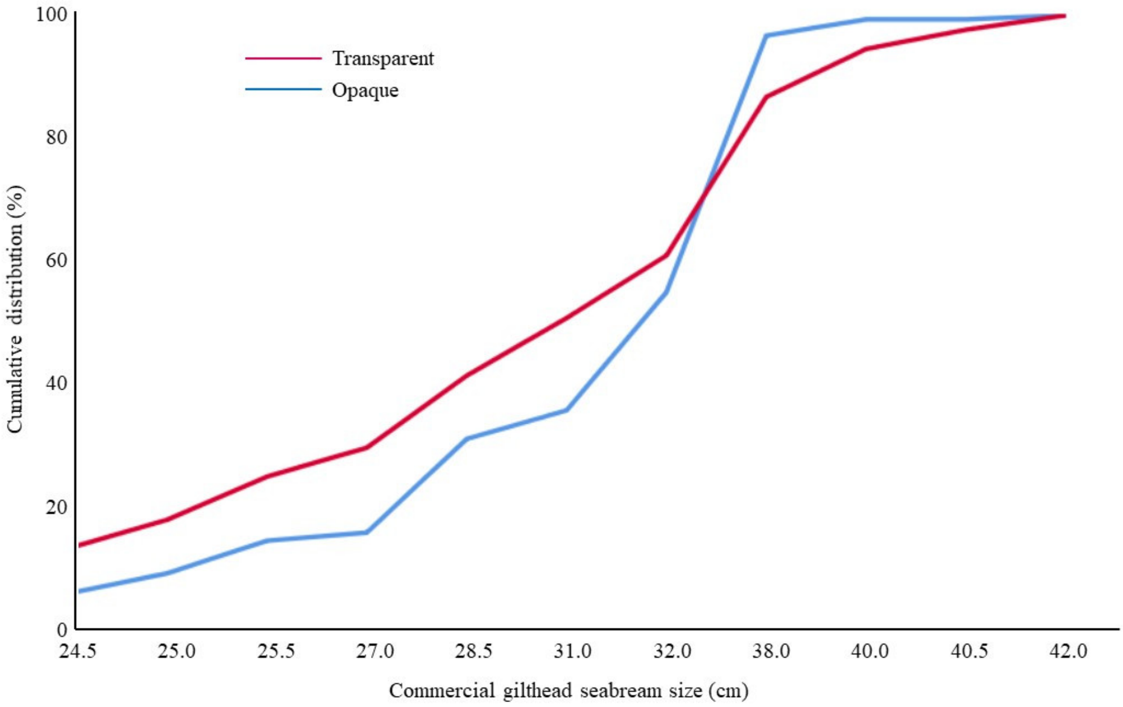

Figure S5. Cumulative distribution (%) of transparent and opaque microplastics according to CGS size.
